# Circulación de *Leishmania infantum* y *Trypanosoma cruzi* en perros domésticos de áreas urbanas de Sincelejo, región Caribe de Colombia

**DOI:** 10.7705/biomedica.6369

**Published:** 2022-12-01

**Authors:** Karol Liseth Rueda-Concha, Ana Payares-Mercado, Jesús Guerra-Castillo, Jesús Melendrez, Yasmit Arroyo-Munive, Lily Martínez-Abad, Suljey Cochero, Eduar Elías Bejarano, Luis Enrique Paternina

**Affiliations:** 1 Grupo Investigaciones Biomédicas, Universidad de Sucre, Sincelejo, Colombia Universidad de Sucre Universidad de Sucre Sincelejo Colombia; 2 Clínica Veterinaria Mascotas, Sincelejo, Colombia Clínica Veterinaria Mascotas Sincelejo Colombia

**Keywords:** Leishmania infantum, Trypanosoma cruzi, zoonosis, urbanización, Colombia, Leishmania infantum, Trypanosoma cruzi, zoonoses, urbanization, Colombia

## Abstract

**Introducción.:**

La enfermedad de Chagas y la leishmaniasis tradicionalmente se han considerado zoonosis endémicas de áreas rurales del país. Sin embargo, la aparición de casos de estas enfermedades en áreas urbanas sugiere nuevos ciclos de circulación de estos parásitos. Por esta razón, se ha propuesto a los perros como centinelas de estos agentes zoonóticos, dado su rol como huéspedes accidentales o reservorios.

**Objetivo.:**

Evaluar la circulación silenciosa de *Leishmania* spp. y *Trypanosoma cruzi* en perros de zonas urbanas de la ciudad de Sincelejo, Sucre.

**Materiales y métodos:**

. Se analizaron 100 muestras de sangre de perros para amplificar la región ITS1 de *Leishmania* spp. Las muestras positivas se utilizaron para amplificar la región conservada del minicírculo del ADN del cinetoplasto de *Leishmania infantum* y para el análisis de polimorfismos de longitud de fragmentos de restricción con la endonucleasa HaelII. Por otra parte, se amplificó un fragmento del ADN satelital de *T cruzi*. Además, se evaluó la presencia de infecciones por *Ehrlichia canis* y *Anaplasma platys*, como potencialmente modificadoras de las manifestaciones clínicas.

**Resultados.:**

De los 100 perros estudiados, se detectó: *Leishmania* spp. en 32, *T*. *cruzi* en 12, ambos parásitos en 7 y *L*. *infantum* en 18. Se encontraron infecciones por anaplasmatáceos en 18, y coinfecciones por bacterias y parásitos en 8 de los perros. En general, 47 de los animales estaban infectados por, al menos, un agente etiológico.

**Conclusión:**

. Se demuestra la circulación de *L. infantum* y *T. cruzi* en zonas urbanas de Sincelejo, así como coinfecciones de estos parásitos junto con parásitos de la familia Anaplasmataceae. El presente estudio demuestra la conveniencia del uso de perros en la vigilancia epidemiológica de estos agentes zoonóticos.

## Abstract (en)

**Introduction:**

*Leishmania infantum* and *Trypanosoma cruzi* are considered endemic zoonotic agents in rural areas of the country; however, there is a high risk of urbanization due to anthropogenic processes. For this reason, dogs have been proposed as sentinels of these zoonoses given their role as patients, hosts and/or reservoirs.

**Objective:**

To assess the silent circulation of *Leishmania* spp. and *Tcruzi* parasites in canines from urban areas of Sincelejo, Sucre.

**Materials and methods:**

One hundred canine blood samples were used to amplify the ITS1 region of *Leishmania* spp. Positive samples were used to amplify the conserved region of the kinetoplast DNA minicircle of *L. infantum* and for restriction fragment length polymorphism analysis with HaelII endonuclease. In addition, a satellite DNA of *T cruzi* was amplified. Also, the presence of *Ehrlichia canis* and *Anaplasma platys* was evaluated as infections that can influence clinical symptoms and health of animals.

**Results:**

*Leishmania* spp. was detected in 32% (32/100) and *Tcruzi* in 12% (12/100) of the animals, and 7% (7/100) of the samples were positive for both parasites. Also, *L. infantum* and infections with Anaplasmataceae family parasites were both detected in 18 % (18/100) of the samples. In the same way, co-infections with bacteria and parasites were found in 8 % (8/100) of the animals. Overall, 47 % (47/100) of the animals were infected with at least one agent.

**Conclusion:**

The circulation of *L. infantum* and *Tcruzi,* as well as co-infections of pathogens of the Anaplasmataceae family, is demonstrated in urban areas of Sincelejo. The present study demonstrates the convenience of canines as epidemiological surveillance sentinels of these zoonotic agents.

Las zoonosis representan un gran porcentaje de todas las enfermedades infecciosas recientemente identificadas, así como de muchas de las ya existentes. Se estima que, aproximadamente, el 61 % de todos los agentes patógenos humanos conocidos son zoonóticos, lo que corresponde a cerca del 75 % de todos los patógenos emergentes durante la última década, y es un problema serio en salud pública humana y veterinaria ^1^.

Entre estas zoonosis, se encuentran la leishmaniasis y la tripanosomiasis americana, conocida también como la enfermedad de Chagas; ambas enfermedades son transmitidas por vectores y causadas por protozoarios parásitos pertenecientes a la familia Trypanosomatidae ^2^. Estas parasitosis se consideran problemas graves de salud pública porque representan un riesgo potencialmente mortal, si no se diagnostican y tratan a tiempo ^3^^,^^4^.

La enfermedad de Chagas es causada por la infección con el parásito *Trypanosoma cruzi*, que afecta entre 6 y 7 millones de personas, principalmente en Latinoamérica, incluyendo a Colombia ^3^^,^^5^. Entre el 2008 y el 2019, se han registrado 21 brotes por transmisión oral en los departamentos de Santander, Casanare, Cesar, Meta, Bolívar, Antioquia y, últimamente, en otros departamentos como Chocó, Atlántico y Sucre ^6^. Los brotes de enfermedad de Chagas ocurridos por transmisión oral han aumentado la tasa de letalidad a 7,9 %, valor que está por encima de otras enfermedades transmitidas por vectores de relevancia en el país, como fiebre por dengue y malaria ^7^.

Por otra parte, la leishmaniasis es causada por diferentes especies del género *Leishmania* que pueden provocar compromiso cutáneo, mucoso o visceral; infortunadamente este último, la leishmaniasis visceral, suele presentarse en niños de zonas donde la enfermedad es endémica. Esta forma clínica es causada por el parásito *Leishmania infantum y* suele ser letal si no se trata a tiempo ^8^.

En Colombia, al final del año 2019, se registraron 237 casos de enfermedad de Chagas y 4.962 casos de leishmaniasis, para un total de 5.199 casos en conjunto. De estos casos, 555 se registraron en los departamentos de la región Caribe de Colombia, distribuidos así: la enfermedad de Chagas en Atlántico, Cesar y Sucre, y la leishmaniasis en Bolívar, Córdoba, Guajira, Magdalena, Sucre, Atlántico, Barranquilla y Cesar, y en las ciudades de Santa Marta y Cartagena ^6^.

En términos cuantitativos, se puede considerar una contribución moderada (10,67 % del total de casos reportados); sin embargo, es necesario mencionar que casi todos los casos de leishmaniasis visceral (cuadro muy letal) en el país se presentan en el núcleo de los departamentos de Córdoba, Sucre y Bolívar ^9^. Estos dos últimos componen el foco mixto de leishmaniasis de los Montes de María.

Tanto la enfermedad de Chagas como la leishmaniasis se han considerado zoonosis endémicas de áreas rurales del país. Sin embargo, desde hace algunos años, se han reportado numerosos hallazgos de parásitos en insectos vectores (flebotomíneos, como *Lutzomyia evansi*, *L. panamensis y L. gomezi*, y triatominos, como *Panstrongylus geniculatus*) en animales sinantrópicos (*Mus musculus y Rattus rattus*) y algunos casos de enfermedad de Chagas en zonas urbanas, principalmente en la región Caribe (Sincelejo, Ovejas y Cartagena) ^10^^-^^17^. (Romero-Ricardo LR, Martínez L, Pérez-Doria A, Bejarano EE. Infección natural de *Panstrongylus geniculatus* con *Trypanosoma cruzi* en el área urbana de Sincelejo, Sucre. XVI Congreso Colombiano de Parasitología y Medicina Tropical. 2015-10-20) Estos hallazgos indican la circulación de ambos parásitos tripanosomatídeos en zonas urbanas de esta región.

En el estudio de ambas enfermedades en Latinoamérica, los perros se han utilizados exitosamente para revelar la circulación de *T cruzi y Leishmania* spp. en áreas endémicas que son principalmente rurales. Esto se debe a que los perros se consideran huéspedes importantes de *T cruzi* en países como Argentina, Venezuela, Panamá, Costa Rica y Colombia, entre otros; además, han sido incriminados como el principal reservorio de *L. infantum* (agente causal de leishmaniasis visceral en América) y se han encontrados infectados con otras especies de *Trypnosoma*^2^^,^^13^^,^^14^^,^^18^^-^^21^.

Debido al papel que pueden desempeñar los perros, ya sea como reservorios, huéspedes accidentales o como centinelas de la transmisión de estos agentes zoonóticos, por su cercanía con las poblaciones humanas y por su constante exposición a vectores de *T cruzi y* de *Leishmania* spp. (triatominos y flebotomíneos, respectivamente), la vigilancia epidemiológica de estos animales puede considerarse una herramienta fundamental para obtener información relevante sobre potenciales eventos de urbanización y prever, incluso, la ocurrencia de brotes de estas enfermedades ^2^^,^^18^^-^^20^.

Dados los antecedentes de ambos parásitos en zonas periurbanas ^21^ y urbanas (Romero-Ricardo LR, Martínez L, Pérez-Doria A, Bejarano EE. Infección natural de *Panstrongylus geniculatus* con *Trypanosoma cruzi* en el área urbana de Sincelejo, Sucre. XVI Congreso Colombiano de Parasitología y Medicina Tropical) de Sincelejo, el objetivo del presente trabajo fue evaluar la potencial circulación de estos parásitos tripanosomátidos en perros de zonas urbanas de la capital del departamento de Sucre.

## Materiales y métodos

### Área de estudio

El estudio se llevó a cabo en el área urbana del municipio de Sincelejo, ciudad capital del departamento de Sucre, el cual pertenece a la subregión de los Montes de María. Se encuentra ubicado a 213 m sobre el nivel del mar, a latitud 9°18'16"N y longitud 75°23'52"O. El clima de la ciudad es tropical seco, con temperatura media anual de 26,6 °C y precipitación anual de 1.164 mm, aproximadamente. El área urbana congrega cerca de 269.000 habitantes (>90 % de la población total del municipio) y el 24,9 % de esta población la constituyen menores entre los 0 y los 14 años ^22^.

### Tipo de estudio y muestra caninas

El estudio fue de tipo exploratorio. Todos los perros participantes asistían regularmente a las clínicas veterinarias por diferentes motivos (como peluquería, guardería, *spa y* consultoría clínica), tenían un plan sanitario activo (con vacunación y desparasitación) y se consideraban residentes de la zona urbana de la ciudad. El personal de la clínica valoró el estado de salud de cada animal, y registró los datos básicos, como edad, sexo, raza (nomenclatura del *American Kennel Club*) y barrio de residencia.

### Registro de datos y toma de muestra de sangre periférica

Se tomaron muestras de 2 ml de sangre periférica por medio de punción de la vena cefálica. Las muestras se depositaron en microtubos de polipropileno (Axygen, USA) con EDTA o BD Tubos K3EDTA Vacutainer™ (Fisher Scientific, Madrid). Cada muestra se rotulada con un código alfanumérico que correspondía con los datos básicos de cada animal incluido en el estudio. Las muestras de sangre total se almacenaron a 4 °C hasta su procesamiento molecular.

### Extracción de ADN genómico y evaluación de su integridad

La extracción de ADN genómico a partir de la muestra de sangre periférica se hizo siguiendo un protocolo con altas concentraciones salinas y usando una solución de cloroformo:alcohol isoamílico (24:1) ^23^. El ADN extraído se resuspendió en 30 pl de agua ultrapura hasta su uso en las pruebas moleculares posteriores.

La integridad de todos los extractos de ADN se evaluó mediante una PCR convencional con los cebadores UNFOR403 (5-TGA GGA CAA ATA TCA TTC TGA GG-3) y UNREV1025 (5-GGT TGT CCT CCA ATT CAT GTT A-3), que permite amplificar una región aproximadamente de 623 pares de bases del gen mitocondrial citocromo b en los mamíferos ^24^.

### Detección de ADN de Leishmania spp. mediante PCR del ITS1 ribosómico

Para detectar el ADN de *Leishmania* spp., se intentó amplificar la región nuclear ITS1 ribosómica empleando los cebadores LITSR (5-CTG GAT CAT TTT CCG ATG-3) y L5.8S (5-TGA TAC CAC TTA TCG CAC TT-3), que amplifican un fragmento entre 300 y 350 pb, según el complejo de *Leishmania* al que pertenezca cada especie detectada: 300 pb para especies del subgénero *L*. (*Viannia*), 317 a 320 pb para especies del complejo *L*. *donovani* como *L*. *infantum, y* 330 a 350 pb para especies del complejo *L. mexicana*^25^.

La mezcla de la reacción tuvo un volumen final de 10 pl, compuesto por: Buffer Mix 5X, Buffer Q Plus 1X, 0,5 μΜ de cada cebador, 1 U de Taq ADN Polimerasa (ExAct TM Plus) y 3 μl de ADN problema.

La amplificación se optimizó con respecto a la temperatura de fusión de los cebadores y se hizo bajo las siguientes condiciones: 3 minutos de desnaturalización inicial a 94 °C, seguida por 40 ciclos de desnaturalización a 94 °C durante 30 segundos, alineamiento de cebadores a 53 °C durante 30 segundos y extensión de la cadena a 72 °C durante 45 segundos, con una extensión final a 72 °C durante 5 minutos. El material amplificado se mantuvo a 4 °C hasta su posterior análisis en geles de agarosa ^26^.

### Detección específica del kADN de Leishmania infantum

La detección específica de ADN de *L*. *infantum* en las muestras que presentaron la amplificación correspondiente al ITS1 ribosómico de *Leishmania* spp., se realizó mediante una segunda PCR, utilizando los iniciadores FLC2 (5’-GTC AGT GTC GGA AAC TAA TCC GC-3’) y RLC2 (5’-GGG AAA TTG GCC TCC CTG AG-3’), que flanquean un segmento de 230 pb de la región conservada del minicírculo del ADN del cinetoplasto de *L*. *infantum.* La mezcla de la reacción se realizó en un volumen final de 10 μl que contenía solución tampón 1X, 0,5 μΜ de cada cebador, 0,6 mM de dNTP, 1,5 mM de MgSO_4_, 1,5 U de Taq polimerasa (Applied Biological Materials Inc., Canada) y 3 μl de ADN.

Las condiciones de ciclado fueron optimizadas con respecto a la temperatura indicada por los cebadores y bajo las siguientes condiciones: desnaturalización inicial a 94 °C durante 3 minutos, seguida de 35 ciclos de 1 minuto a 94 °C, luego 1 minuto a 58 °C y finalmente 1 minuto a 72 °C. Después de 10 minutos a 72 °C, el material amplificado se mantuvo a 4 °C hasta el análisis en geles de agarosa ^27^.

### *Identificación de Leishmania* spp. *mediante RFLP y secuenciación de ADN*

Con el fin de confirmar los resultados obtenidos con las estrategias basadas en la amplificación de fragmentos de genes específicos, los productos de PCR de ITS1 de *Leishmania* spp. se sometieron a un análisis de polimorfismos de longitud de fragmentos de restricción (RFLP) con la endonucleasa HaelII, para determinar si los parásitos detectados pertenecían al *L*. *infantum*, al complejo *L*. *mexicana* o a especies del subgénero *L*. (*Viannia*) ^28^^-^^32^, siguiendo las condiciones y recomendaciones de Céspedes-Chaves, *et al.*^33^.

Algunos productos de interés de la ITS1-PCR se seleccionaron para enviarlos a secuenciación (Macrogen, Seúl, Corea del Sur). Las secuencias consenso, obtenidas mediante ensamblaje de los electroferogramas de secuenciación editados, se compararon con las secuencias registradas en las bases de datos GenBank, mediante BLASTn con el algoritmo MegaBLAST. La identificación de especies se confirmó mediante los dendrogramas de las secuencias, utilizando el *software* MEGA X ^34^.

### Detección específica de ADN de Trypanosoma cruzi

El ADN de *T cruzi* se detectó mediante dos PCR distintas. Primero, se amplificó un fragmento de 166 pb del ADN satelital del parásito, utilizando los cebadores Cruzi 1 (5’-AST CGG CTG ATC GTT TCG A-3’) y Cruzi 2 (5’-AAT TCC TCC AAG CAG CGG ATA-3’). Las reacciones se llevaron a cabo en volúmenes de 10 pl que contenían solución tampón de PCR 1X, 1,5 mM de MgSO4, 0,5 μΜ de cada cebador, 1,5 U de Taq Polimerasa (Applied Biological Materials Inc., Canada), 0,6 μl de dNTP y 3 μl de ADN problema. Se utilizó un termociclador Veriti™ (Applied Biosystems, USA) con el siguiente perfil térmico: 3 minutos de desnaturalización inicial a 94 °C, seguido de 40 ciclos a 94 °C durante 30 segundos, 60 °C durante 30 segundos y 72 °C durante 1 minuto. Por último, se hizo una extensión final a 72 °C durante 5 minutos ^35^.

Posteriormente, se practicó una segunda PCR para confirmar los hallazgos en la reacción anterior. En esta reacción se amplificó un fragmento de 330 pb del minicírculo del kADN de *T*. *cruzi* con los cebadores 121 (5’- AAA TAA tGt ACG GGK GAG ATG CAT GA-3’) y 122 (5’- GGT TCG ATT GGG GTT GGT GTA ATA TA-3’). Las reacciones se realizaron en volúmenes de 10 μl, con la siguiente composición: solución tampón de PCR 1X, 1,5 mM de MgSO4, 0,5 μΜ de cada cebador, 1,5 U de Taq Polimerasa (Applied Biological Materials Inc., Canada), 0,6 ul de dNTP y 3 μl de ADN problema. El perfil térmico empleado fue el siguiente: 1 ciclo de desnaturalización inicial a 95 °C por 2 minutos, seguido de 40 ciclos a 95 °C por 20 segundos, 64 °C por 1 minuto, y 72 °C por 1 minuto, y una extensión final de 72 °C por 5 minutos; se empleó el termociclador Applied Biosystems™ Veriti ^36^^,^^37^.

La utilización de ambos tipos de dianas nucleares, ADN satélite y cinetoplásticas, se considera apropiada para el diagnóstico de este parásito en sangre de insectos vectores. Una muestra se consideraba positiva para la infección cuando se obtenía la amplificación de ambas dianas ^38^^,^^39^.

### Detección de Anaplasmataceae transmitidos por garrapatas

Debido a la gran incidencia de infecciones por proteobacterias de la familia Anaplasmataceae (*Anaplasma platys* y *Ehrlichia canis*) en perros del país y a la influencia que pueden ejercer sobre la gravedad de los síntomas que suelen relacionarse con la leishmaniasis canina ^40^^-^^42^, se decidió incluir el tamizaje de ambos parásitos en los perros analizados.

Primero, se hizo la detección específica de *A*. *platys*, empleando los cebadores Apla-Hs475F (5’-AAG GCG AAA GAA GCA GTC TTA-3’) y Apla- Hs1198R (5’-CAT AGT CTG AAG TGG AGG AC-3’) que amplifican una región de 723 pb del gen *groESL* (^43^^-^^45^. Por otra parte, la detección de *E. Canis* fue realizada con los cebadores Ehr-groESLF (5’-CAA TAG CAA GAG CCA ATG- 3’) y Ehr-groESLR (5’-TTA GAA GAT GCT GTA GGA TG-3’) que amplifican una región ~145 pb del operon groESL de este género bacteriano ^46^.

Además, se realizó otra PCR para detectar ADN de *E. canis*, empleando cebadores desarrollados para el presente trabajo denominados: Ecaj_0423F (5-TCC TGT TGA TGG ACA AAG TGT T-3) y Ecaj_0423R (5-ACC TGT AGC ACC ACA TTC TTG-3), que amplifican un fragmento de 403 pb del gen putativo Ecaj_0423 (CP000107).

La diana seleccionada (Ecaj_0423, código de secuencia de 744 nucleótidos) está presente tanto en la cepa Jake de Estados Unidos de América (CP000107) como en la cepa YZ-1 de China (CP025749) y, de acuerdo con los análisis de similitud local contra todas las secuencias depositadas en Genbank (base de datos “nr”), es una región exclusiva de *E*. *canis*. Los cebadores diseñados se evaluaron extensamente en las plataformas InSilico (Universidad del País Vasco) y Primer-BLAST (NCBI) con el fragmento deseado exclusivamente en *E*. *canis*.

### Análisis de datos y aspectos éticos

Los datos de los animales (sexo, raza, edad, y comuna de procedencia) se contrastaron con la infección por los dos parásitos cinetoplástidos mediante pruebas de múltiples proporciones de Fisher o de asociación de ji al cuadrado en el programa R V. 4.1.1 ^47^. Además, utilizando el programa QGIS™, V. versión 3.4 ^48^, se hizo un mapeo de los resultados positivos para *Leishmania*, *T*. *cruzi y* especies de la familia Anaplasmataceae (*Ehrlichia canis, Anaplasma platys* o ambos), de acuerdo con los barrios de procedencia de los animales afectados, con el fin de ilustrar su distribución geográfica.

La toma de muestras solo llevó a cabo con la expresa autorización y firma del consentimiento informado por parte del propietario de cada animal; documentos que reposan en la Clínica para evitar conflictos éticos debido al manejo de datos en la relación médico-cliente-paciente en la práctica veterinaria ^49^. Los informes de los resultados moleculares se remitieron a los médicos veterinarios para concertar el manejo y tratamiento de los animales con los propietarios, aplicar medidas de protección contra vectores (collares impregnados con deltametrina y formulaciones insecticidas o repelentes de uso tópico) y hacer el seguimiento clínico; estas directrices han sido ampliamente recomendadas por expertos en el área ^44^^,^^50^. Todos los procedimientos de toma de muestras, manejo de los animales y tratamiento de los datos fueron avalados por el Comité Institucional de Bioética de la Universidad de Sucre (Sesión Ordinaria N°4, 12 de octubre de 2017).

## Resultados

### Características de la muestra canina analizada

Se incluyeron 100 perros domésticos del sitio de estudio. Se categorizaron en 21 razas (los animales mestizos fueron tratados como una raza para conveniencia del análisis categórico); la más frecuente fue mestizos (19 %; 19/100), seguida de *French Poodle* (15 %; 15/100) y *Pinscher* miniatura (13 %; 13/100). Las edades estuvieron en un rango entre los 3 y los 168 meses. Con respecto al grupo etario, el 70 % (70/100) se consideraron adultos (animales con edades entre los 13 y 84 meses), el 18 % (18/100), seniles (mayores de 84 meses), y el 12 % (12/100), cachorros (animales con edades entre 0 y 12 meses) (cuadro 1).

**Cuadro 1:** Evaluación de la asociación entre variables registradas y número de positivos a *Leishmania* (L+), T *cruzi* (Tc+) y parásitos de la familia Anaplasmataceae (ATG+), en perros residentes en el área urbana de Sincelejo. Los valores de probabilidad (p), en negrilla, se consideraron estadísticamente relevantes (p<0,05).

Variable	Categorías	Muestra	L+	p	Tc+	p	ATG+	p
Sexo	Hembra	50	16	1	5	0,758	14	0,019
	Macho	50	16		7		4	
Grupo etario	Adulto	70	25	0,381	9	0,898	16	0,114
	Cachorro	12	2		1		0	
	Senil	18	5		2		2	
Raza	*Beagle*	2	0	0,701	0	0,810	0	0,906
	*Boston Terrier*	2	0		0		0	
	*Bulldog francés*	6	3		1		1	
	*Bulldog inglés*	1	0		0		0	
	*American Bully*	1	0		0		0	
	*Chow Chow*	1	0		0		0	
	*Cocker*	2	0		0		1	
	*Dogo argentino*	1	1		0		0	
	*Golden*	2	0		0		0	
	*Husky*	6	3		1		1	
	*Labrador*	4	1		0		1	
	*Mestizo*	19	9		3		2	
	*Pincher*	13	3		3		4	
	*Pitbull*	1	0		0		1	
	*Pointer*	1	0		1		0	
	*French Poodle*	15	4		1		2	
	*Pug*	5	2		0		1	
	*Rottweiler*	1	0		0		0	
	*Schnauzer*	7	4		1		1	
	*Shih Tzu*	4	1		1		1	
	*Yorky*	6	1		0		2	
Comuna	C2	14	5	0,279	4	0,670	2	0,806
	C3	19	3		3		2	
	C4	19	9		0		4	
	C5	13	3		0		4	
	C6	3	0		4		1	
	C7	28	10		0		5	
	C8	3	1		0		0	
	C9	1	1				0	

Mediante el examen clínico, se encontró que el 78 % de los perros estudiados fueron asintomáticos y el 22 % restante presentó, al menos, un signo clínico (oligosintomático) indicativo o que se podía relacionar con tripanosomiasis canina, leishmaniasis canina o ambas. En total, se registraron hasta 13 signos clínicos en los 22 animales sintomáticos (solo en la comuna 6 no se presentaron sintomáticos), con la siguiente frecuencia: vómito grave ^4^, diarrea ^3^, fiebre ^3^, sangrado nasal ^2^, sospecha de hemoparásitos ^2^, anemia ^1^, dermatitis ^1^, dermopatía grave ^1^, falla renal ^1^, inapetencia ^1^, infección urinaria ^1^, queratoconjuntivitis seca ^1^ y úlcera corneal ^1^.

En relación con la distribución de los perros en la ciudad, toda la población analizada pertenecía a la zona urbana de Sincelejo y se pudieron representar 8 de las 9 comunas existentes dentro del municipio (comuna 1, sin datos). El mayor número de animales muestreados pertenecía a la comuna 7, seguida de la comuna 3 y la 4 (cuadro 1).

### *Frecuencia de infección por Leishmania* spp*.*

**Figura 1:**
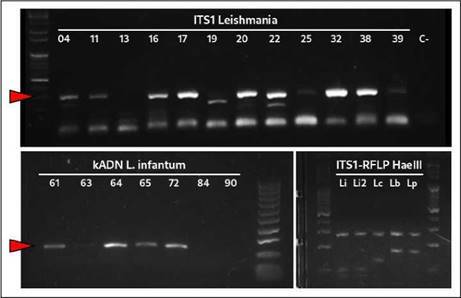
Electroforesis en geles de agarosa al 1,5 % de la PCR para *Leishmania* spp. (ITS1, panel superior). En los paneles inferiores, se observa, específicamente, kADN de *L*. *infantum* (izquierda), así como los perfiles de restricción de ITS1 con HaeIII para cepas de referencia de *L*. *infantum* (Li y Li2), *L*. *colombiensis* (Lc), *L*. braziliensis (Lb) y L. *panamensis* (Lp) (derecha).

La frecuencia de infecciones por *Leishmania* spp., determinada mediante la detección de la región ITS1 ribosómica en la población de perros estudiados, fue del 32 % (32/100) (figura 1). De estos, 16 eran hembras y 16 machos, con edades que oscilaban entre los 7 y los 168 meses (78,2 % de los 32 fueron adultos). La mayoría de los perros infectados por estos parásitos eran mestizos (9 %), seguidos por *French Poodle* (4 %) y por *Schnauzer* (4 %) (cuadro 1). En relación con los signos clínicos, 7 (21,87 %) animales presentaban, al menos, un signo clínico relevante de la infección: 3 de ellos con vómito grave (3/4), uno con úlcera corneal (1/4), uno con dermatopatía (1/4), uno con infección urinaria (1/4) y uno con cuadro febril (1/3).

### *Tipificación de Leishmania* spp*. en los perros infectados*

A partir de las 32 muestras positivas para la ITS1 de *Leishmania* spp., fue posible detectar específicamente ADN de los minicírculos del cinetoplasto de *L. infantum* en 13 animales (40,6 %) (figura 1). Uno de estos animales murió con un cuadro grave de dermatopatía, semanas después de la toma de la muestra en el centro veterinario.

**Figura 2:**
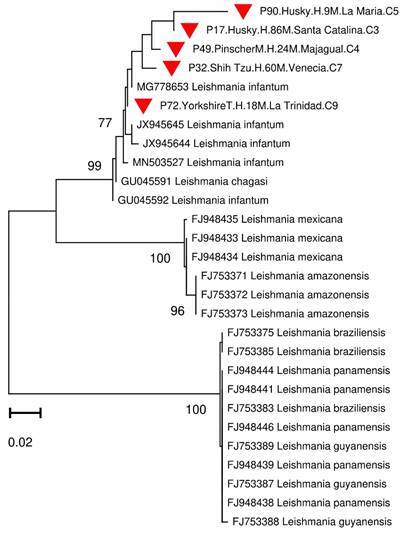
Dendrograma *neighbor-joining* (distancias K2P) de comparaciones pareadas de ITS1 de cepas de *Leishmania spp.* obtenidas de perros de la zona urbana de la ciudad de Sincelejo (triángulo rojo). Los códigos de estas secuencias presentan información adicional del perro, como raza, sexo, edad (en meses), barrio y comuna de procedencia.

Mediante la RFLP, se logró determinar la presencia del perfil de restricción típico para *L*. *infantum* en 11 de las 32 muestras evaluadas (figura 1). Además, se logró visualizar perfiles mixtos que no permitieron la identificación precisa de otras especies*.* Por otro lado, los análisis bioinformáticos de cinco secuencias provenientes de muestras previamente identificadas como *L*. *infantum* (todas determinadas mediante RFLP y 4 de ellas positivas para el kADN de *L*. *infantum*), permitieron confirmar estos resultados con alta confidencia de agrupación (Bootstrap 99) al compararlas con secuencias de referencia de distintas especies de este género (figura 2). Todas las secuencias presentaron una similitud mayor del 95 % y hasta del 100 %, con secuencias de *L*. *infantum* reportadas en Genbank (códigos MN503527-MN496380). De los 18 caninos infectados con *L*. *infantum*, 7 presentaban algún síntoma de los mencionados anteriormente y 11 resultaron completamente asintomáticos.

### Frecuencia de infección por T. cruzi

**Figura 3:**
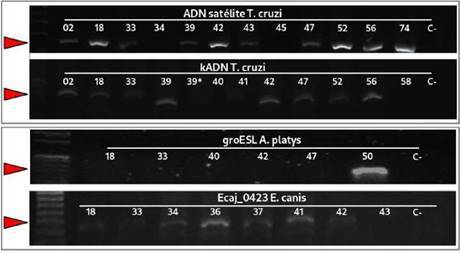
Electroforesis en geles de agarosa al 1,5 % de productos de PCR del tamaño esperado para *Trypanosoma cruzi* (ADN satélite y kADN, panel superior) y PCR específicas para agentes transmitidos por garrapatas, *Anaplasma platys y Ehrlichia canis* (panel inferior)

En cuanto a la frecuencia de infección por *T cruzi*, se pudo determinar que el 12 % de las 100 muestras evaluadas fueron positivas en las pruebas practicadas, empleando los dos juegos de cebadores diseñados para la detección del ADN esta especie (figura 3). De estos, 5 fueron hembras y 7 machos, con un rango de edad entre los 7 y los 144 meses. La mayor frecuencia de infección con *T*. *cruzi* se obtuvo en perros adultos (9 %; 9/100). Además, 8 de las 21 razas representadas en la muestra presentaron, al menos, un individuo infectado, siendo los mestizos y los *Pincher* los más afectados por estos parásitos (3 %; 3/100). Cabe resaltar que cuatro de los perros infectados presentaron síntomas: uno de ellos con vómito grave, uno con infección urinaria, otro con úlcera corneal y otro con fiebre.

Además, se encontraron 7 perros coinfectados con parásitos del género *Leishmania* spp. (4 de específicamente con *L*. *infantum* y *Tcruzi*) y, en 2 de ellos, se encontraron signos clínicos de úlcera corneal y, en otro, infección urinaria.

### *Frecuencia de infección por proteobacterias de la familia* Anaplasmataceae

En relación con la infección por especies de la familia Anaplasmataceae (*Ehrlichia canis, Anaplasma platys* o ambos), 18 animales resultaron infectados con *E*. *canis y* uno de ellos presentó coinfección con *A*. *platys* (figura 3). De estos, 5 presentaron síntomas: 2 con fiebre, 1 con vómito grave, 1 con falla renal y uno con infección urinaria.

En este tamizaje, 10 perros estuvieron infectados exclusivamente con especies de la familia Anaplasmataceae (*Ehrlichia canis, Anaplasma platys* o ambos): 3 con coinfección con *Leishmania* spp. (todos por *L*. *infantum y* uno con coinfección con *A*. *platys y E*. *canis*), 3 con *T*. *cruzi* (todos *E*. *canis*), y finalmente, 2 con infección múltiple por especies de la familia Anaplasmataceae (*E. canis*), *T cruzi y L. infantum*. En general, 47 animales dieron positivo, al menos, para uno de los agentes evaluados, siendo el 50 % de estos asintomáticos (11/22).

### Distribución de animales infectados y análisis de variables

En todas las comunas estudiadas, se encontraron animales infectados con alguno de los cuatro agentes analizados (*Leishmania*, *T*. *cruzi*, *E*. *canis* o *A*. *platys*). Las comunas 4 y 7 presentaron la mayor cantidad de estos (13 cada una), mientras que solo un positivo fue hallado en las comunas 6, 8 y 9.

En cuanto a los parásitos, se encontraron animales positivos para *L. infantum* en todas las comunas analizadas, a excepción de la comuna 6; hubo 3 animales en la comuna 2, 2 en la 3, 3 en la 4, 2 en la 5, 6 en la 7, 1 en la 8, y 1 en la comuna 9.

**Figura 4:**
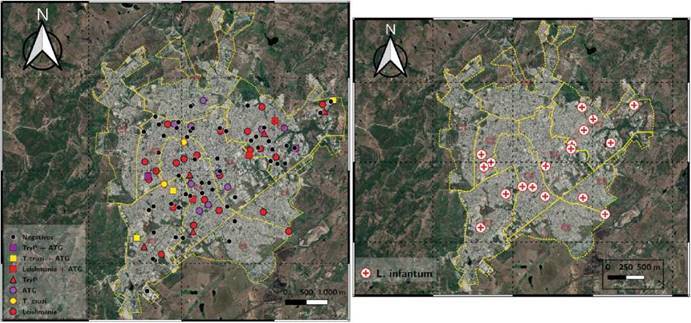
A la izquierda, distribución espacial de parásitos tripanosomátidos y anasplasmatáceos detectados en caninos y a la derecha, distribución de *Leishmania infantum* en perros residentes de la zona urbana del municipio de Sincelejo.

En el caso de *T cruzi*, este fue hallado en 4 de las 7 comunas: 1 en la Comuna 2, 4 en la Comuna 3, 3 en la Comuna 4, y 4 en la Comuna 7. En cuanto a *T cruzi*, se encontró en 4 de las 7 comunas, así: uno en la comuna 2, 4 en la 3, 3 en la 4, y 4 en la 7. Todos los resultados mencionados anteriormente se ilustran espacialmente de acuerdo con las divisiones por comunas de la ciudad de Sincelejo (figura 4).

No se encontró asociación estadísticamente significativa al contrastar los resultados positivos de la PCR para parásitos del género *Leishmania y T*. *cruzi*, con las características de la población estudiada de perros (edad, sexo, raza y comuna de residencia).

## Discusión

La distribución de los casos de leishmaniasis y tripanosomiasis americana en humanos y animales domésticos ha cambiado con el desarrollo urbanístico de los asentamientos humanos, así como por factores sociales e industriales a escala global ^51^. Los ciclos de transmisión de estas enfermedades se consideran influenciares por actividades humanas, como la migración de personas desde áreas rurales hacia áreas urbanas, y otros fenómenos como la creciente deforestación y el cambio climático, los cuales pueden inducir, a su vez, cambios en la ecología de los vectores y los reservorios de los microorganismos patógenos ^52^.

Anteriormente, la importancia del perro doméstico en el ciclo de transmisión de *T*. *cruzi y L*. *infantum* en Colombia estuvo restringida al ámbito rural ^53^^,^^54^. Sin embargo, desde hace algunos años se ha sugerido que estos animales podrían estar implicados en los procesos de urbanización de estos parásitos; por lo tanto, la vigilancia epidemiológica de los perros (como centinelas) en estas áreas, se considera fundamental para comprender su participación en estas “nuevas dinámicas” de transmisión ^2^^,^^55^.

La circulación de parásitos del género *Leishmania* en Sincelejo está íntimamente relacionada con la cercanía a zonas endémicas, principalmente los Montes de María, uno de los focos mixtos de leishmaniasis más importantes en Colombia. La facilidad de movilización de la población humana y animal, desde zonas rurales o hacia aquellas donde la enfermedad es endémica, permite el desplazamiento de animales infectados; esto se suma a la presencia y gran abundancia de los vectores en esta zona del país, lo que favorece que se mantenga el ciclo de transmisión de estos parásitos en áreas urbanas ^10^^,^^56^^,^^57^. Todos estos se consideran factores que predisponen a la circulación de agentes zoonóticos como *L*. *infantum*, capaces de afectar a una gran parte de la población infantil de la ciudad, dada su amplia distribución en la zona urbana y, por supuesto, la presencia del vector local ^58^. Estos hallazgos encienden las alarmas sobre la potencial aparición de casos autóctonos de leishmaniasis visceral, aunque es necesario aclarar que aún no existen datos sobre vectores infectados por *Leishmania* spp. en la ciudad.

La frecuencia de las infecciones por *Leishmania* spp. en perros en zonas urbanas de Sincelejo (32 %; 32/100), es tan alta como la reportada por Paternina, *et al.,* hace 8 años en zonas rurales de este municipio ^21^; estos autores encontraron que el 34,9 % (29/83) de los perros de Sabanas del Potrero (zona rural y periurbana de Sincelejo) estaban infectados con parásitos del género *Leishmania,* lo que sugiere la potencial y eventual aparición de ciclos de transmisión urbanos, y que los caninos estén participando, probablemente, como reservorios o huéspedes de estos parásitos ^21^.

Además, se ha encontrado que la frecuencia de infección es cercana a la reportada (44 %; 52/118) en perros de zonas urbanas del municipio de Ovejas, departamento de Sucre; aunque esta frecuencia es mayor a la observada en el presente trabajo, vale la pena señalar que este municipio, a diferencia de la ciudad de Sincelejo, reporta periódicamente casos urbanos de leishmaniasis humana y pertenece al área que se considera endémica en Los Montes de María ^59^.

Las grandes frecuencias de infección reportadas en perros de zonas urbanas de Sincelejo podrían sugerir su papel como reservorio de parásitos. Sin embargo, los xenodiagnósticos en perros indican que los animales asintomáticos o con pocas manifestaciones clínicas no son fuentes de parásitos tan eficientes para la infección con *Lutzomyia evansi* (vector local), en comparación con los perros con múltiples síntomas ^60^^,^^61^.

En cuanto al diagnóstico de las 18 muestras identificadas como *L*. *infantum* (utilizando combinación de RFLP, PCR de kADN o secuenciación de ADN), 4 se identificaron mediante las tres técnicas usadas para la tipificación de *Leishmania* spp. Con esto se confirma la circulación de este parásito en el área urbana de Sincelejo, aportando evidencias de la urbanización de estos parásitos en diversas zonas de la ciudad. Su presencia en Sincelejo se suma a los reportes sobre otras áreas urbanas del Caribe colombiano: primero en Cartagena (Región Caribe), en un perro con diagnóstico serológico de este mismo parásito ^17^, luego en el área urbana del municipio de Ovejas (Región Caribe), en cultivos de ganglio poplíteo e isoenzimas ^62^; por otra parte, se suma el reporte de un caso de leishmaniasis canina por *L*. *infantum* en el área urbana de Cali (Región Andina), confirmado mediante serología, RFLP y secuenciación de ADN ^63^.

Con relación a la positividad de *T cruzi*, curiosamente, es superior a la reportada en Cumaral (Meta), donde se reportó en 1,6 % de los perros ^19^. En contraste, estos hallazgos se consideran similares a los reportados en las zonas urbanas de cuatro municipios del departamento de Santander con frecuencias de infección de 12,1 % ^64^, y a lo reportado en un área rural de Boyacá, donde se encontraron frecuencias de infección del 15 % ^65^.

En cuanto a la circulación de este parásito en Sincelejo, la frecuencia de infección en estos animales puede deberse a transmisión vectorial por presencia de triatominos infectados (Romero-Ricardo LR, Martínez L, Pérez- Doria A, Bejarano EE. Infección natural de *Panstrongylus geniculatus* con *Trypanosoma cruzi* en el área urbana de Sincelej o, Sucre. XVI Congreso Colombiano de Parasitología y Medicina Tropical), aunque no se descarta la vía transfusional y, especialmente, la oral. Esta última fue responsable de un brote reciente, al menos, de 15 casos (3 de ellos fatales) en zona rural del municipio El Roble (departamento de Sucre) ^6^^,^^66^. Por esta razón, el hallazgo de estos parásitos en perros de zonas urbanas puede considerarse muy preocupante.

En general, se determinó que 7 perros estaban coinfectados con *Leishmania y T cruzi*, 4 de ellos específicamente por *L. infantum y T cruzi*, siendo este el primer estudio en reportar esta coinfección en perros en un centro urbano del país.

En Latinoamérica, esta coinfección solo se ha reportado en perros de zonas endémicas para ambas enfermedades: Venezuela ^2^ y Brasil ^67^^,^^68^.

La interacción entre *Leishmania y Trypanosoma* en un mismo huésped puede alterar la frecuencia u ocurrencia de la infección y la patogenicidad, como suele ser evidente por la presentación de síntomas como la onicogrifosis y las lesiones cutáneas ^67^. Además, la presencia de coinfecciones puede influir en el diagnóstico clínico y alterar la respuesta a los tratamientos, e influir potencialmente en la dinámica de transmisión por medio de la capacidad infecciosa de los vectores, como se ha demostrado anteriormente ^61^^,^^69^.

El diagnóstico de infección por especies de la familia Anaplasmataceae (en particular, *E. canis*) se considera relevante debido a que se ha asociada previamente con leishmaniasis canina. En nuestro caso, aunque es frecuente en perros, solo 3 de los 8 animales coinfectados por especies de la familia Anaplasmataceae y algún o ambos tripanosomátidos, fueron sintomáticos: uno con fiebre, uno con vómito grave y otro con infección urinaria.

De hecho, se puede inferir que las infecciones por especies de la familia Anaplasmataceae se podrían encontrar en fase crónica debido a la gran cantidad de asintomáticos (13 de 18 positivos para especies de la familia Anaplasmataceae) y su incidencia principalmente en adultos. La asociacion entre la positividad a especies de la Familia Anaplasmataceae y las hembras es fácilmente explicable, debido a la composicion de la muestra, puesto que las comunas 4, 5 y 7 aportaron 34 de las 50 hembras al analisis y de las 14 hembras infectadas, 11 provenían de estas 3 comunas lo que explica la tendencia a un mayor número de hembras infectadas.

Con base en los resultados obtenidos, se demuestra la circulación de *L*. *infantum y T*. *cruzi* en perros domésticos de zonas urbanas de la ciudad de Sincelejo, independientemente de la infección por *E. canis y A. platys*. No existió ninguna relación entre las especies de la familia Anaplasmataceae y los tripanosomatídeos evaluados. Los hallazgos demuestran la utilidad de los caninos como centinelas en la vigilancia de estas zoonosis, los cuales alertan sobre la eventual aparición de brotes de ambas enfermedades en centros urbanos del Caribe.
